# *Meis1*: effects on motor phenotypes and the sensorimotor system in mice

**DOI:** 10.1242/dmm.030080

**Published:** 2017-08-01

**Authors:** Aaro V. Salminen, Lillian Garrett, Barbara Schormair, Jan Rozman, Florian Giesert, Kristina M. Niedermeier, Lore Becker, Birgit Rathkolb, Ildikó Rácz, German Mouse Clinic Consortium, Martin Klingenspor, Thomas Klopstock, Eckhard Wolf, Andreas Zimmer, Valérie Gailus-Durner, Miguel Torres, Helmut Fuchs, Martin Hrabě de Angelis, Wolfgang Wurst, Sabine M. Hölter, Juliane Winkelmann

**Affiliations:** 1 Institute of Neurogenomics, Helmholtz Zentrum München, 85764 Neuherberg, Germany; 2 Institute of Developmental Genetics, Helmholtz Zentrum München, 85764 Neuherberg, Germany; 3 German Mouse Clinic, Institute of Experimental Genetics, Helmholtz Zentrum München, 85764 Neuherberg, Germany; 4 German Center for Diabetes Research (DZD), 85764 Neuherberg, Germany; 5 Institute of Molecular Animal Breeding and Biotechnology, Gene Center, Ludwig-Maximilians-University München, 81377 Munich, Germany; 6 Institute of Molecular Psychiatry, Medical Faculty, University of Bonn, 53127 Bonn, Germany; 7 Chair of Molecular Nutritional Medicine, Technical University Munich, EKFZ – Else Kröner Fresenius Center for Nutritional Medicine, Gregor-Mendel-Str. 2, 85350 Freising-Weihenstephan, Germany; 8 Department of Neurology, Friedrich-Baur-Institute, Klinikum der Ludwig-Maximilians-Universität München, Ziemssenstr. 1a, 80336 Munich, Germany; 9 Deutsches Zentrum für Neurodegenerative Erkrankungen e. V. (DZNE), 81377 Munich, Germany; 10 Munich Cluster for Systems Neurology (SyNergy), Adolf-Butenandt-Institut, Ludwig-Maximilians-Universität München, 81377 Munich, Germany; 11 Centro Nacional de Investigaciones Cardiovasculares (CNIC), 28029 Madrid, Spain; 12 Chair of Experimental Genetics, School of Life Science Weihenstephan, Technische Universität 85354 Freising, Germany; 13 Chair of Developmental Genetics, Faculty of Life and Food Sciences Weihenstephan, Technische Universität München, 85354 Freising, Germany; 14 Institute of Human Genetics, Klinikum Rechts der Isar, Technische Universität München, 81675 Munich, Germany; 15 Neurologic Clinic, Klinikum rechts der Isar, Technische Universität München, 81675 Munich, Germany

**Keywords:** Meis1, Prepulse inhibition, Restless legs syndrome, Sensorimotor system, Mouse model, Pramipexole

## Abstract

*MEIS1* encodes a developmental transcription factor and has been linked to restless legs syndrome (RLS) in genome-wide association studies. RLS is a movement disorder leading to severe sleep reduction and has a substantial impact on the quality of life of patients. In genome-wide association studies, *MEIS1* has consistently been the gene with the highest effect size and functional studies suggest a disease-relevant downregulation. Therefore, haploinsufficiency of *Meis1* could be the system with the most potential for modeling RLS in animals. We used heterozygous *Meis1*-knockout mice to study the effects of *Meis1* haploinsufficiency on mouse behavioral and neurological phenotypes, and to relate the findings to human RLS. We exposed the *Meis1*-deficient mice to assays of motor, sensorimotor and cognitive ability, and assessed the effect of a dopaminergic receptor 2/3 agonist commonly used in the treatment of RLS. The mutant mice showed a pattern of circadian hyperactivity, which is compatible with human RLS. Moreover, we discovered a replicable prepulse inhibition (PPI) deficit in the *Meis1*-deficient animals. In addition, these mice were hyposensitive to the PPI-reducing effect of the dopaminergic receptor agonist, highlighting a role of Meis1 in the dopaminergic system. Other reported phenotypes include enhanced social recognition at an older age that was not related to alterations in adult olfactory bulb neurogenesis previously shown to be implicated in this behavior. In conclusion, the *Meis1*-deficient mice fulfill some of the hallmarks of an RLS animal model, and revealed the role of Meis1 in sensorimotor gating and in the dopaminergic systems modulating it.

## Abstract (precis)

**Summary:** Loss of *Meis1* results in motor restlessness in mice, a phenotype resembling human restless legs syndrome, as well as altered sensorimotor gating and improved social discrimination memory.

## INTRODUCTION

*MEIS1* encodes a TALE homeobox transcription factor known to play a role in hematopoiesis and vascular patterning (Azcoitia et al., 2005; Carramolino et al., 2010; Hisa et al., 2004), as well as in the development of the proximodistal limb axes (Mercader et al., 1999), the nervous system (Barber et al., 2013; Spieler et al., 2014) and the development of various organs, such as the heart (Mahmoud et al., 2013), eyes (Marcos et al., 2015) and pancreas (Zhang et al., 2006). *MEIS1* cooperates with other transcription factors to perturb myeloid differentiation in leukemogenesis (Nakamura et al., 1996) and, in genetic association studies, it has been linked with restless legs syndrome (RLS) (Winkelmann et al., 2007, 2011), periodic leg movements during sleep (Moore et al., 2014; Stefansson et al., 2007), symptoms of insomnia (Lane et al., 2016), PR interval (Butler et al., 2012) and waist-to-hip ratio (Shungin et al., 2015). Structurally, MEIS1 consists of a Pbx interaction motif and a TALE homeodomain near the C-terminus, responsible for DNA interaction. It has two major functional splice variants, MEIS1A and MEIS1B, which diverge in the C-terminus of the protein (Huang et al., 2005). These isoforms are differentially expressed during development and are likely to differ in their genomic targets and effects on neural differentiation (Maeda et al., 2001). MEIS1 binds transcription start sites and genomic enhancers alone and in interaction with *PBX* and *HOX* proteins, regulating the expression of genes involved in the processes of axon guidance, protein phosphorylation and cell differentiation, among others (Marcos et al., 2015; Penkov et al., 2013).

RLS is a sensorimotor movement disorder causing severe sleep loss and impaired quality of life (Trenkwalder and Paulus, 2010). The hallmark symptoms include an urge to move the legs, relieved by voluntarily moving the legs. The symptoms are predominantly present in the evening and night time, and at rest. Epidemiological evidence shows that RLS is more common in females compared to males (Berger and Kurth, 2007), but the sex difference could be explained by the number of previous pregnancies (Pantaleo et al., 2010). In the context of RLS, *MEIS1* contributes to the heritability of the disease through both common and rare genetic variation. In genome-wide association studies (GWAS), *MEIS1* has consistently been the gene with the largest effect size, with an odds ratio up to 1.74 (Winkelmann et al., 2007, 2011). In large-scale sequencing and family studies, putative causal loss-of-function rare variants have been identified (Schulte et al., 2014). A *MEIS1* intronic haplotype linked to RLS risk is associated with decreased expression of MEIS1 (Xiong et al., 2009). Therefore, *Meis1* haploinsufficiency could be considered the most promising animal model system for human RLS. *Meis1* knockout mice, in which *Meis1* has been inactivated by knocking in a modified ERT2 domain (*Meis1^tm1Mtor^*), die during development due to a fetal hematopoietic failure (Azcoitia et al., 2005). Heterozygous animals, however, show a hyperactive phenotype potentially compatible with components of human RLS (Spieler et al., 2014). In addition, the mutant animals show low fertility, low body mass at birth and various eye-related phenotypes (Azcoitia et al., 2005; Marcos et al., 2015).

In a bid to provide a more comprehensive understanding of the effect of *Meis1* haploinsufficiency on the central nervous system, we exposed these heterozygous *Meis1-*deficient mice to a series of motor, sensorimotor and cognitive behavioral and neurological tests. To further disentangle what role this gene plays in the brain and how it could be involved in RLS, we also determined the effect of a commonly used RLS therapeutic dopaminergic receptor 2/3 (D2/3R) agonist, pramipexole, as well as performing concomitant semi-quantitative measures of dopaminergic neuron number, iron metabolism, believed to play an important role in the pathogenesis of RLS, and adult neurogenesis, the birth and assimilation of new neurons.

## RESULTS

### *Meis1* haploinsufficiency leads to sex-dependent motor alterations at middle age

We have shown previously (Spieler et al., 2014) that *Meis1* haploinsufficiency leads to increased locomotor activity in the open field in both male and female young adult mutant mice at the age of 9 weeks. Furthermore, these animals were hyperactive as indexed by a tendency towards increased locomotor activity over a 21-h period in the home-cage environment of PhenoMaster cages (Spieler et al., 2014). In the current study, we determined whether the severities of these effects are maintained with age. Thus, we assessed the same locomotor activity parameters in middle-aged mice (9-11 animals per genotype and sex) from the age of 38-40 weeks.

**Fig. 1.**
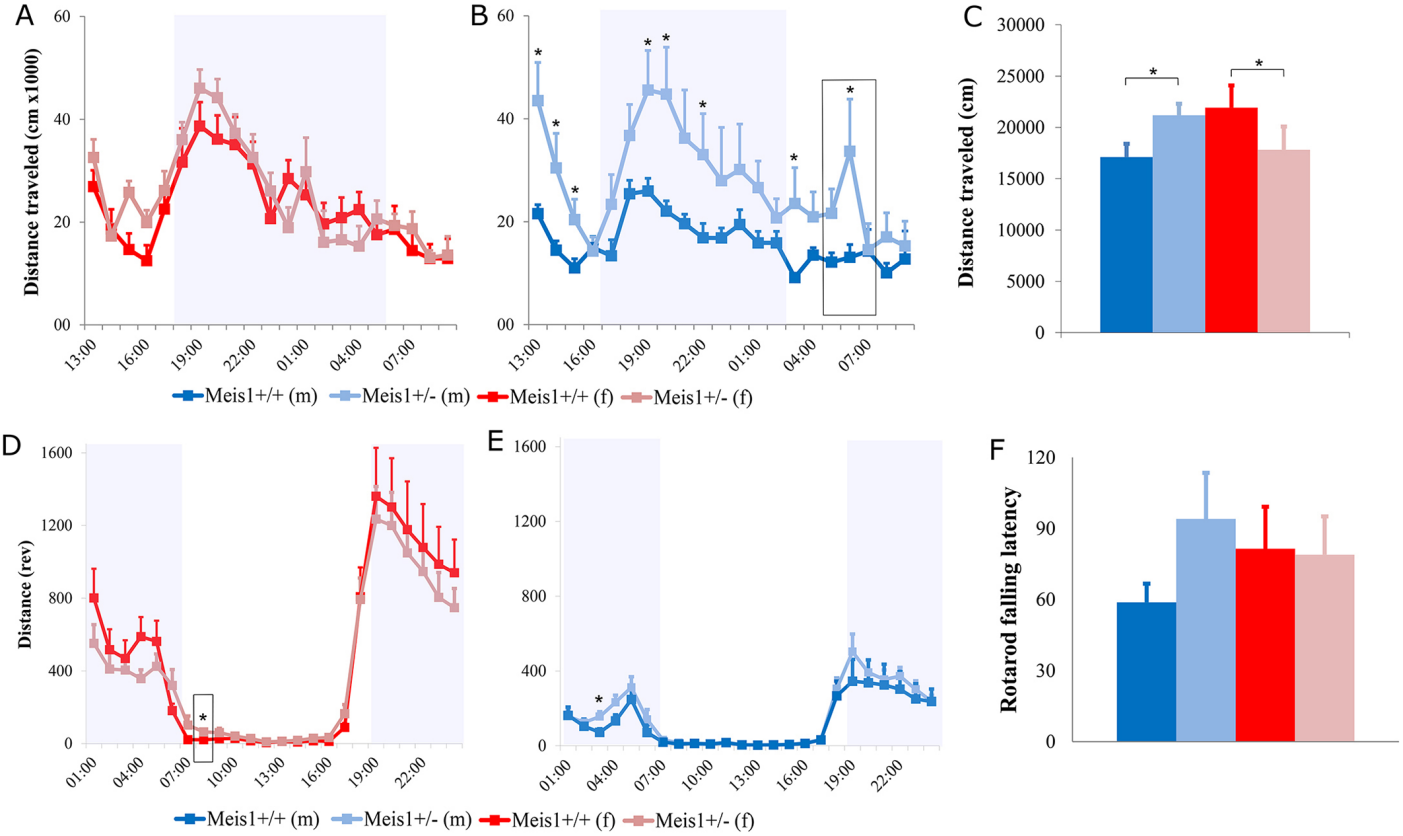
**Measures** **of locomotor activity, motor coordination and balance.** Measures of locomotor activity in the PhenoMaster cages (females: A, males: B), open field (C) and voluntary running wheels (females D, males E), and motor coordination and balance in the rotarod (F; seconds). *P<0.05 vs +/+ controls. (B,D) Boxes indicate an increase in activity just after lights on in the rest phase seen in female +/− mice in voluntary running wheels (D) and in male +/− mice in home-cage activity (B). The cohort included 21 female (9 +/− and 12 +/+) and 23 male (10 +/− and 13 +/+) mice. Results are plotted as means±s.e.m. rev, revolutions.

At this older age, *Meis1* haploinsufficiency leads to locomotor alterations that dichotomize based on sex. This was manifest as a significant locomotor increase in the male mutant mice in the open field, which was not evident in the females (2-way ANOVA, sex×genotype interaction effect: *F*_1,43_=5.64, *P*=0.022, Fig. 1C). Locomotor activity alterations in response to the open field could reflect an anxiety reaction to the novelty of the arena even though this was not evident in the anxiety-related index of center time in the open field (2-way ANOVA, sex×genotype interaction effect: *F*_1,43_=0.16, *P*=0.69, genotype effect: *F*_1,43_=0.12, *P*=0.72, sex effect: *F*_1,43_=0.83, *P*=0.37). Thus, we sought to establish whether this hyperactivity in the male mutant mice could be confirmed with longer-term observation in the PhenoMaster cages where the mice are permitted to habituate to the surroundings. It is then possible to see whether the locomotor differences are also present when exploration is the principal behavior exhibited by the mice during their active phase and whether it is subject to circadian variation, as would be expected from an RLS animal model. With this analysis, we again saw that *Meis1* haploinsufficiency caused increased locomotor activity in middle-aged males and this was evident over several 1-h time bins, most importantly in the beginning of the inactive phase (1-way ANOVA, genotype effect: *F*_1,21_=4.92, *P*=0.038, Fig. 1B). By dividing the 21-h period into sub-phases it transpired that the male mutant mice were hyperactive on first transfer to the PhenoMaster cages during the habituation phase (first 3 h). Control levels of activity were then attained until after lights off, when the male mutant mice again showed higher activity during the active phase. The activity levels in the control male mice continued to decrease until lights on. Nevertheless, the male mutant mice showed another peak in activity at the start of the lights-on phase, when the controls were at rest. The effects of *Meis1* haploinsufficiency in the female mice on home-cage locomotor activity was less pronounced (Fig. 1A). During the last hour of the habituation phase, the female mutant mice showed increased activity compared to controls, without major differences throughout the remainder of the testing period.

As an additional form of locomotor activity analysis, we measured voluntary wheel running within the home cage (Fig. 1D,E). Each mouse was given access to a running wheel, voluntary activity was recorded continuously and the data was averaged over 21 days to yield a 24-h activity profile. *Meis1* haploinsufficiency in the male mice did not produce differences in the amount or pattern of voluntary running-wheel activity (Fig. 1E). Nevertheless, the female mutant mice, although not exhibiting marked deviations from the control profile, showed a specific increase in activity during the first hour of the lights-on phase, when the control mice are at rest (Fig. 1D).

As a measure of motor coordination and balance we also assessed latency to fall from the rotarod. There was a small tendency for the male mutant mice to show increased latencies to fall from the rod without clear differences in the females (2-way ANOVA, sex×genotype interaction effect: *F*_1,39_=1.431, *P*=0.239, genotype effect: *F*_1,39_=0.133, *P*=0.294, sex effect: *F*_1,39_=0.145, *P*=705).

Together with our previously published findings (Spieler et al., 2014), these data indicate that *Meis1* haploinsufficiency causes sex-, age- and situation-dependent effects on motor restlessness in mice.

### *Meis1* deficiency causes sensorimotor gating deficits without effects on thermal sensitivity

**Fig. 2.**
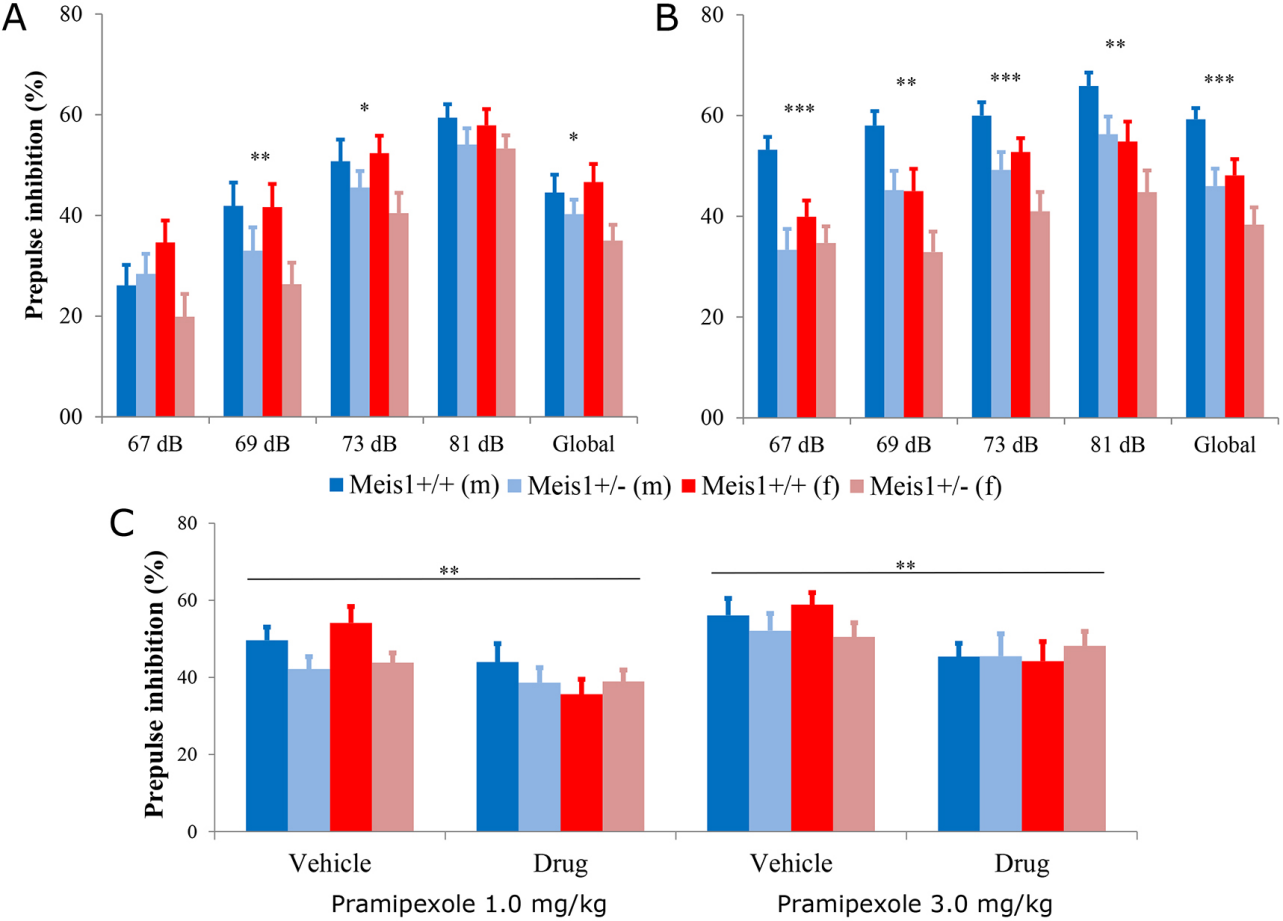
**Prepulse inhibition (PPI).** The PPI results from the first screening cohort (A, *n*=15 per sex per genotype) and the replication cohort (B, *n*=15 per sex per genotype). The results show that the *Meis1* knockout genotype effect on PPI was replicable when compared to wild-type littermates. Results are plotted as means±s.e.m. Genotype effects: *P<0.05, ** *P*<0.005, ***P<0.001 calculated by two-way ANOVA with sex as a factor, including interaction term. (C) The global effect of pramipexole 1.0 mg/kg body weight (left) and 3.0 mg/kg body weight (right) on the PPI of wild-type and heterozygous animals is shown. Results are plotted as means±s.e.m. Genotype effects: **P<0.005 calculated by two-way ANOVA with sex as a factor, including interaction term.

An alteration in thermal sensitivity has been shown in human patients with secondary RLS, and intense unpleasant feelings in the legs characterize the disease (Bachmann et al., 2010). It was thus germane to assess sensory perception consequent to *Meis1* haploinsufficiency. As a measure of thermal sensitivity, we assessed the effect of *Meis1* haploinsufficiency in the hotplate test in mice at the younger age of 11 weeks. There were no significant nociceptive differences detected between the genotype groups in licking or shaking behavior, or response times (Table 1). At the age of 10 weeks, the animals were also tested for acoustic startle reactivity (ASR) and prepulse inhibition (PPI) of the acoustic startle reflex. ASR was decreased in male heterozygous mutant mice with the opposite effect in female mutants compared to controls (repeated measures ANOVA: sex×genotype×dB interaction effect, *P*<0.001) (Fig. S1). PPI was decreased in mutant mice of both sexes at 69 dB (2-way ANOVA, genotype effect: *F*_1,56_=7.132, *P*=0.01, sex effect: *F*_1,56_=0.577, *P*=0.45, sex×genotype interaction effect: *F*_1,56_=0.504, *P*=0.48) and 73 dB (2-way ANOVA, genotype effect: *F*_1,56_=5.04, *P*=0.03, sex effect: *F*_1,56_=0.206, *P*=0.65, sex×genotype interaction effect: *F*_1,56_=0.767, *P*=0.38), as well as globally across all prepulse intensities (2-way ANOVA, genotype effect: *F*_1,56_=5.79, *P*=0.019, sex effect: *F*_1,56_=0.228, *P*=0.63, sex×genotype interaction effect: *F*_1,56_=1.233, *P*=0.27, Fig. 2A). It was possible to replicate the effect of *Meis1* deficiency on PPI in an independent cohort of mice at the age of 9 weeks, where again a decrease in PPI was observed  at 69 dB (2-way ANOVA, genotype effect: *F*_1,60_=10.575, *P*=0.002, sex effect: *F*_1,60_=10.88, *P*=0.0016, sex×genotype interaction effect: *F*_1,60_=0.009, *P*=0.93), 73 dB (2-way ANOVA, genotype effect: *F*_1,60_=12.227, *P*=0.001, sex effect: *F*_1,60_=5.74, *P*=0.020, sex×genotype interaction effect: *F*_1,60_=0.024, *P*=0.88) and 81 dB (2-way ANOVA, genotype effect: *F*_1,60_=7.198, *P*=0.009, sex effect: *F*_1,60_=9.47, *P*=0.0031, sex×genotype interaction effect: *F*_1,60_=0.004, *P*=0.95) as well as globally (2-way ANOVA, genotype effect: *F*_1,60_=13.723, *P*<0.001, sex effect: *F*_1,60_=9.14, *P*=0.0037, sex×genotype interaction effect: *F*_1,60_=0.313, *P*=0.58) in both sexes (Fig. 2B). The effect on ASR was not observed in this cohort (the opposite effect was found) and was thus not replicable. The assessment of ASR and PPI was not possible in the middle-aged cohort owing to natural age-related sensorineural hearing loss, associated with the genetic background used. The hearing loss was not specific to either genotype group, but was seen in all mice. There were no clear differences in auditory brainstem responses (ABRs) in the mice of cohort 1 at 17 weeks (Table 1).

**Table 1.**
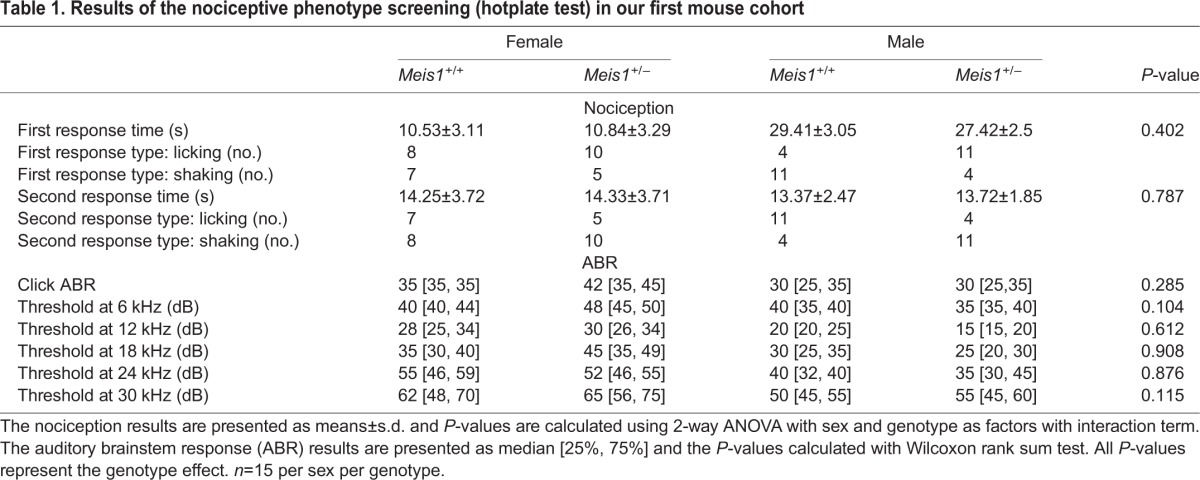
**Results of the nociceptive phenotype screening (hotplate test) in our first mouse cohort**

### *Meis1* deficiency confers insensitivity to D2/3R-agonist effects on sensorimotor gating

Given this robust and replicable effect of *Meis1* haploinsufficiency on sensorimotor gating ability, we wanted to determine the effect of a dopaminergic receptor agonist used in the treatment of RLS on this phenotype. Thus, the effect of pramipexole, a preferential D2/3R agonist, at two doses [1 and 3 mg/kg bodyweight intraperitoneal (i.p.)] on PPI was assessed with saline vehicle control. In the vehicle-treated mice, the mutant animals showed a higher reactivity to acoustic startle than the wild types at higher sound intensities. PPI was decreased in the mutant mice after vehicle injection, replicating again the findings of the first two cohorts. The 1 mg/kg dose of pramipexole had an effect at high sound intensities in the mutant group, making their startle response even stronger. In the control group, the startle response was only altered at lower sound intensities. The 3 mg/kg dose of pramipexole, on the other hand, increased the startle response of the wild-type mice at higher stimulus intensities but had no effect on the mutant group. The results are shown in Fig. S1.

PPI was affected by pramipexole in a more consistent manner. In the wild-type group, PPI was significantly reduced by both high and low doses of pramipexole. The PPI in these animals was reduced to the level of the vehicle-treated mutant mice. In contrast, in the heterozygous mutants, the PPI was not significantly altered by pramipexole (Fig. 2C).

**Fig. 3.**
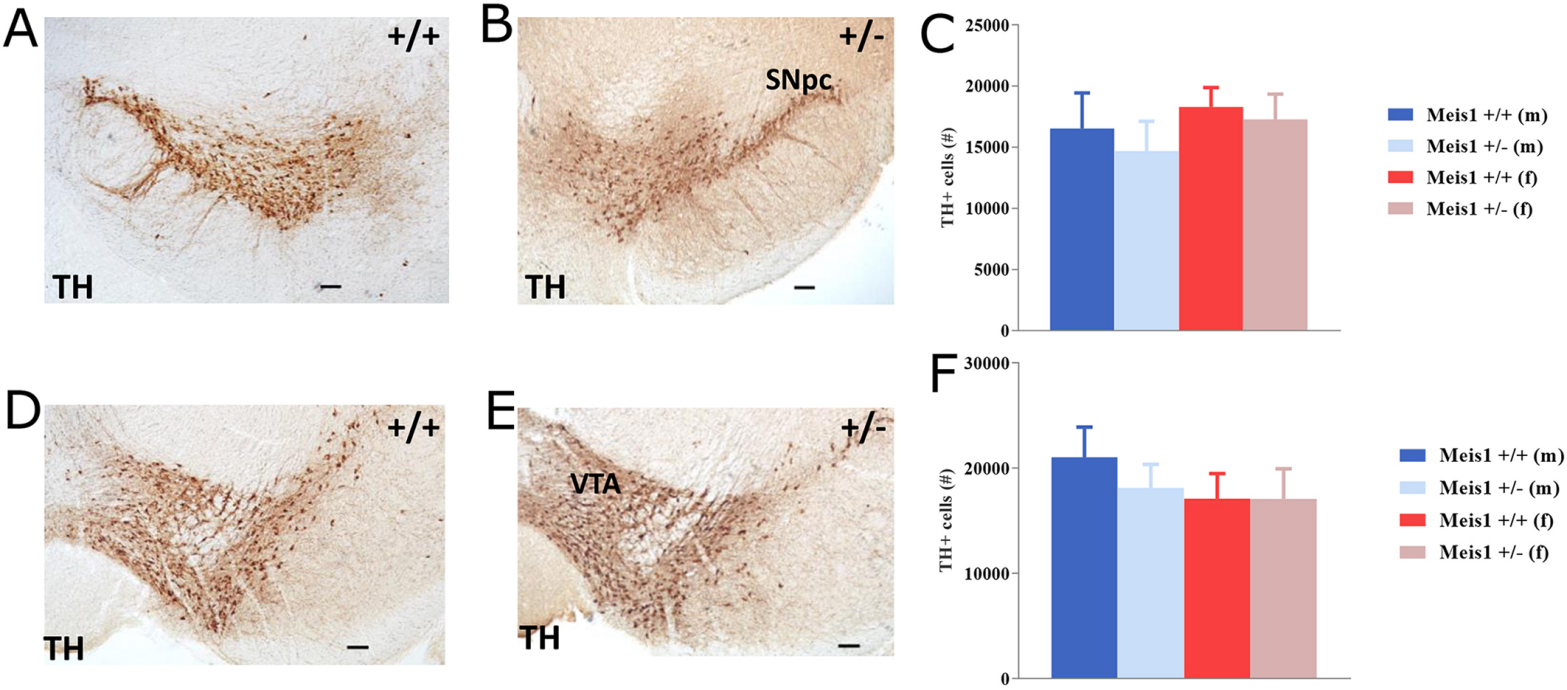
**Dopaminergic neurons.** Tyrosine hydroxylase (TH)+ neurons in the substantia nigra pars compacta (SNpc; A,B) and ventral tegmental area (VTA; D,E) of wild-type (+/+) and heterozygous knockout (+/−) mice. Scale bars: 100 µm. (C,F) Results are plotted as means±s.e.m. No differences were found between the groups in either the SNpc (C) or VTA (F). Groups are *n*=3 male +/+, *n*=4 male +/−, *n*=4 female +/+, *n*=5 female +/−.

To investigate whether this insensitivity to the D2/3R agonist pramipexole was influenced by the number of dopaminergic neurons, which could lead to altered D2/3R signaling, using optical fractionator estimates we quantified the number of tyrosine hydroxylase (TH)-positive cells in the substantia nigra pars compacta (SNpc) and the ventral tegmental area (VTA). We did not observe clear differences in the total number of TH+ cells in either the SNpc (2-way ANOVA, genotype effect: *F*_1,12_=0.401, *P*=0.538, Fig. 3C) or the VTA (2-way ANOVA, genotype effect: *F*_1,12_=0.291, *P*=0.599, Fig. 3F) between genotypes to indicate that neuron number differences lead to the altered signaling. Furthermore, we assessed iron homeostasis by measuring the plasma levels of iron, ferritin (iron storage) and transferrin (iron binding) (Table 2) because iron has been suggested to play a major role in RLS pathogenesis in humans (Earley et al., 2014). There were no clear differences between the groups in any of the three measures except for a tendency towards a decrease in the iron-storage protein ferritin in the female *Meis1*-deficient mice (2-way ANOVA, sex×genotype interaction effect: *F*_1,56_=3.32, *P*=0.074).

**Table 2.**
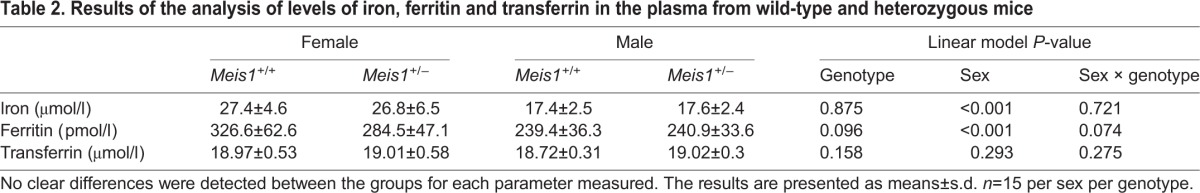
**Results of the analysis of levels of iron, ferritin and transferrin in the plasma from wild-type and heterozygous mice**

### *Meis1* haploinsufficiency alters social memory

**Fig. 4.**
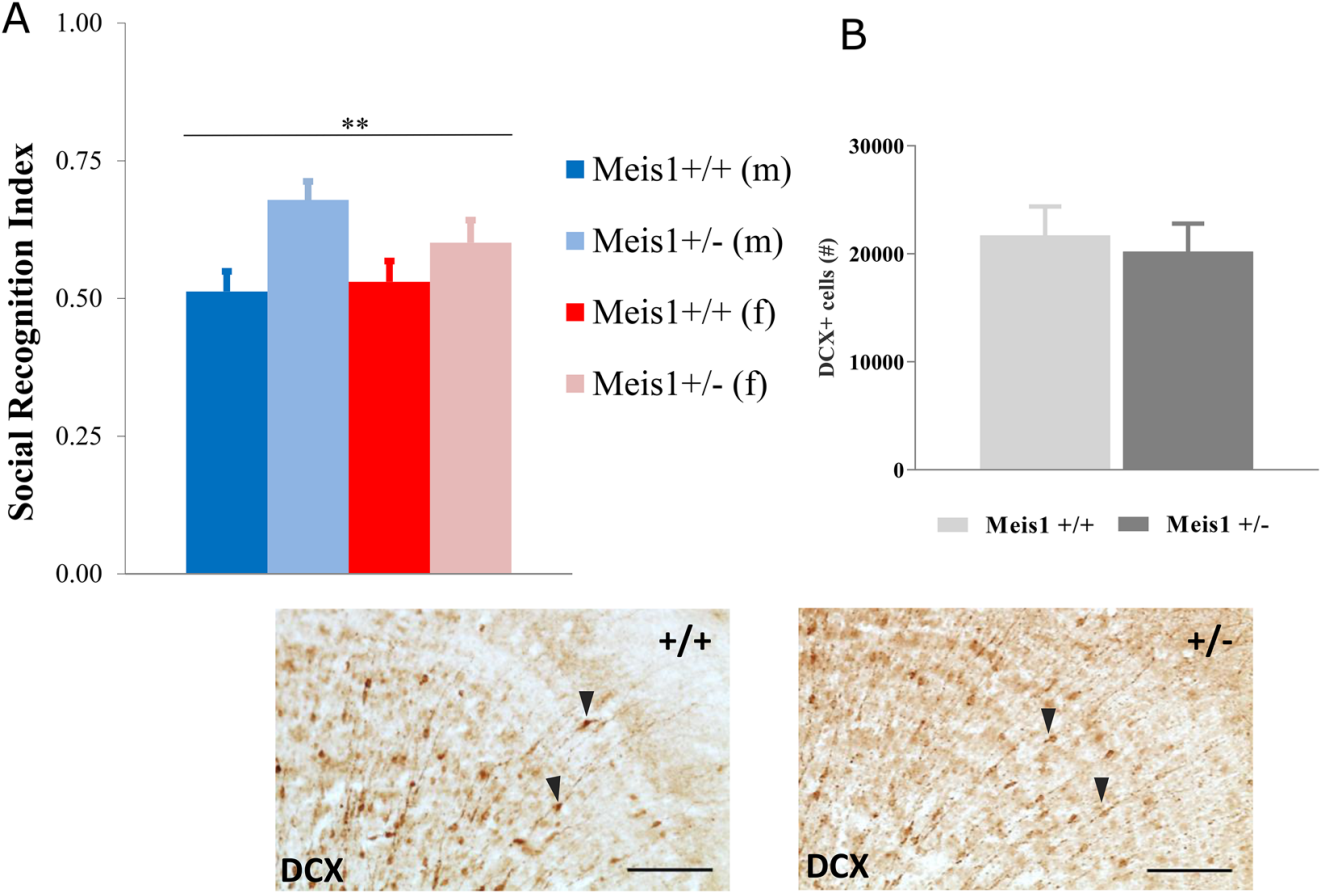
**Behavioral alterations are not associated with modifications of adult neurogenesis in *Meis1*-deficient mice.** (A) Social recognition test revealed an increased social recognition index in the mutant animals compared to wild types. The cohort included 21 female (9 heterozygous and 12 wild-type) and 23 male (10 heterozygous and 13 wild-type) mice. **P<0.01, 2-way ANOVA genotype effect: *F*_1,41_=9.48, *P*=0.004, *Meis1*^+/+^ vs *Meis1*^+/−^. (B) No clear differences were detected in the level of adult neurogenesis in the olfactory bulb granular cell layer (OB) of the mutant mice as indexed by the number of doublecortin (DCX)+ cells in this region. Data from males and females were pooled for this analysis (*n*=6 +/+, *n*=6 +/−). Results are plotted as means±s.e.m. Representative photomicrographs are also depicted showing DCX+ cells (black arrowheads) in the OB of wild type (+/+) and heterozygous mutant (+/−) mice. Scale bars: 100 µm.

The olfactory social recognition index was increased in both female and male mutant mice compared to wild types (Fig. 4A; 2-way ANOVA, genotype effect: *F*_1,41_=9.48, *P*=0.004, sex effect: *F*_1,41_=0.58, *P*=0.45, sex×genotype interaction effect: *F*_1,41_=1.45, *P*=0.24), suggesting better social discrimination memory. There were no genotype-related differences in social investigation time during the sample phase of the test (data not shown). The social recognition index showed no correlation with distance traveled in the open field (*r*=−0.050, *P*=0.749), with time spent in the middle of the arena (*r*=−0.166, *P*=0.282) or with total activity in the running wheel (*r*=−0.073, *P*=0.679). None of the parameters showed significant correlation when the sexes were tested separately (data not shown). We have shown previously that decreased social memory can be due to decreased adult neurogenesis in mice (Garrett et al., 2015). Thus, to determine whether the behavioral alterations were associated with modifications of the adult neurogenesis in *Meis1*-deficient mice, a stereological approach was used to quantify the number of doublecortin (DCX)-positive cells in the granular cell layer of the olfactory bulb (Fig. 4B). DCX expression is an immature neuron marker in the adult brain that has also been shown to reflect overall levels of neurogenesis, and DCX+ cell quantification can be used as an alternative to BrdU pulse/chase analysis (Couillard-Despres et al., 2005). Optical fractionator estimates showed that there was no significant effect of genotype on the number of DCX+ cells in the granular cell layer of the olfactory bulbs in these mice (unpaired *t*-test: *t*_10_=0.401, *P*=0.697).

## DISCUSSION

Haploinsufficiency of *Meis1* is a potential model system for simulating RLS in mice. Here, we show that this Meis1 deficit causes a sex- and context-dependent RLS-related phenotype at middle age, impaired sensorimotor gating ability that was refractory to the D2/3R agonist pramipexole as well as increased social memory. These behavioral abnormalities occurred without differences in thermal sensitivity, plasma iron concentrations or dopaminergic neuron number in the SNpc/VTA. Thus, although *Meis1* haploinsufficiency causes the RLS-related phenotype of motor restlessness in mice, the detailed nature of these effects is complex. Furthermore, it is characteristically distinct from other genetic RLS models (e.g. *Btbd9* knock-out), highlighting the heterogeneous nature of the effects of these different risk genotypes in this disease.

Motor restlessness in these *Meis1*-haploinsufficient mice was detected previously in young adult male and female mice (Spieler et al., 2014). RLS is, however, more prevalent at an older age in humans (Berger et al., 2004) and therefore we also tested middle-aged mice. Human RLS occurs in a circadian fashion where the patients are typically affected in the evening hours, and therefore a circadian hyperactivity pattern specific to the beginning of the inactive period should be expected from an RLS animal model. So, although we have shown here that the overall locomotor effects in the open field and PhenoMaster cages deviate per the sex of the animal in middle age, both the male and female *Meis1*-haploinsufficient mice still show such an RLS-like phenotype: specific increases in activity during the initial stage of the rest phase. In the males, this occurs in the PhenoMaster cages and, in the females, in their voluntary wheel-running activity pattern. In males, however, hyperactivity is also observed during the active period, making the effect less specific to the RLS-relevant time bins. *Meis1*-haploinsufficiency thus leads to RLS-like circadian motor restlessness in middle age that, in the case of the females, requires amplification with an additional environmental stimulus. The latter could represent the urge to move, a key feature of human RLS. This outcome further reveals that RLS associated with *Meis1* risk haplotypes is likely to produce sexually dimorphic motor effects in middle age.

In terms of sensory function, the *Meis1* deficiency did not alter thermal sensitivity in response to the hotplate, which is incongruous with the effects in the *Btbd9* mouse RLS model (DeAndrade et al., 2012). Nevertheless, augmented thermal sensitivity is a feature of patients with secondary RLS, whereas patients with idiopathic RLS experience different types of sensitivity (Bachmann et al., 2010). We also report here the first evidence to indicate that a Meis1 deficit leads to a reproducibly decreased PPI response. This deficit occurs in the absence of ABR alterations, suggesting that the PPI phenotype is not secondary to impaired hearing in the mice. PPI of the acoustic startle reflex is a commonly used rodent readout for sensorimotor gating and integration. Impairment of PPI has been observed in neurological disorders (Kohl et al., 2013) and it is most frequently used as a readout in schizophrenia research (Swerdlow and Light, 2015). PPI was not altered in the single study conducted in RLS patients so far (Leon-Sarmiento et al., 2015). However, the patients were not stratified by their *MEIS1* genotype in that study and it is possible that PPI is a phenotype specific to RLS associated with *Meis1* risk haplotypes. This prospect is also supported by the lack of PPI differences in homozygous *Btbd9*-knockout mice (*Btbd9^tm1b(EUCOMM)Wtsi^*) (Meehal et al., 2017; see also: www.mousephenotype.org/data/experiments?geneAccession=MGI:1916625). Thus, PPI should be studied in RLS patients who also are carriers of the *MEIS1* risk allele.

In our treatment experiment, pramipexole, a D2/3R agonist, did not rescue the PPI phenotype in *Meis1*-deficient mice. On the contrary, it reduced the PPI in wild-type mice to the level of the mutants. The effect of pramipexole on wild-type mice contradicts an earlier study reporting no pramipexole effect on PPI in C57BL/6 mice (Chang et al., 2010). However, the *Meis1*-deficient mice were bred on a C57BL/6JOlaHsd substrain background and this different outcome could be explained by the high strain sensitivity of the dopaminergic effect on PPI (Swerdlow et al., 2004). The lack of effect on the mutant animals might indicate that the *Meis1*-deficient mice are hyposensitive to pramipexole, which may signify the involvement of Meis1 in the dopaminergic system. It has been demonstrated in neuropathological studies that there is a higher level of TH, the dopaminergic synthetic enzyme, in the substantia nigra of RLS sufferers and fewer D2 receptors in the putamen correlated with RLS severity (Connor et al., 2009). Thus, given that PPI is regulated by dopaminergic systems of the nucleus accumbens and striatum (Swerdlow et al., 2001), to test whether it may be a causative factor in the insensitivity to pramipexole in these mice we quantified the number of TH+ neurons in the SNpc and VTA. Nevertheless, our results did not indicate significant differences in the numbers of dopaminergic neurons in these areas. It remains possible that the expression of dopamine receptors is reduced in *Meis1*-deficient mice and this will require further study. There were also no differences in plasma iron homeostasis. This again distinguishes the *Meis1*-haploinsufficient mice from the *Btbd9* mice, where the latter also shows decreased serum iron levels (DeAndrade et al., 2012). Nevertheless, the *Btbd9* mice did not show differences in brain-tissue iron levels. Thus, it would be propitious to quantify striatal iron levels in these *Meis1*-deficient mice.

We have shown here, for the first time, a non-sensorimotor cognitive phenotype associated with *Meis1* deficiency. The *Meis1*-deficient mice had improved social memory as indexed by enhanced social recognition ability. There is limited conflicting evidence available concerning the cognitive ability of RLS patients with reports of deficits (Kim et al., 2014), enhanced ability (Moon et al., 2014) and no alterations in different types of memory (Driver-Dunckley et al., 2009; Fulda et al., 2010). These inconsistencies could relate to the type of memory assessed, the age and sex of the patient, the type of RLS (idiopathic versus secondary), medication status and, as previously alluded, patients in these studies have not been stratified according to risk haplotypes. Moon and colleagues have shown that drug-naïve idiopathic RLS patients, predominantly at middle age, exhibit enhanced verbal fluency and word memory (Moon et al., 2014). They conjectured that this increased ability may be an adaptation to sleep disturbance manifesting as heightened alertness with an unknown underlying mechanism. We have demonstrated previously involvement of adult neurogenesis in social discrimination memory in the *DCXCreER^T2^; DTA* model where loss of DCX+ cells and hence decreases in adult neurogenesis reversibly impaired social recognition memory (Garrett et al., 2015). Therefore, given the impaired social recognition memory, we hypothesized that abnormal neurogenesis in the olfactory bulbs of *Meis1*-deficient mice may underlie this deficit. Our quantification of DCX+ cells in the olfactory bulb did not, however, support this hypothesis and no differences were observed. This indicates that alternative processes likely mediate the enhanced social memory consequent to Meis1 deficiency. Recent evidence showing zonal Meis1 expression in the main olfactory epithelium could signify one potential avenue for further investigation in this regard (Parrilla et al., 2016). Furthermore, in light of this new evidence, a more detailed analysis of cognitive ability in RLS patients with the *MEIS1* risk haplotype is warranted.

In summary, *Meis1*-deficient mice show an RLS-like motor-restlessness phenotype, a sensorimotor gating deficiency refractory to a D2/3R agonist and some heretofore unknown *Meis1-*deficiency-related effects. Other proposed RLS-related genetic mouse models such as *Btbd9* (DeAndrade et al., 2012) and *Ptprd* (Drgonova et al., 2015) exhibit degrees of motor restlessness. There are features of *Meis1*-deficient mice, however, that are distinct and could be explained by the genetic architecture of the disease. For example, when a motor phenotype (periodic leg movements during sleep) is used to assess RLS in human genetic studies, the *BTBD9* locus has the highest effect size (Moore et al., 2014; Stefansson et al., 2007). When the sensory RLS symptoms are assessed, the *MEIS1* locus is the top hit (Winkelmann et al., 2007, 2011). This indicates that the specific disease characteristics in patients will depend on the combination of risk alleles at different genetic risk loci and this is supported by the current data. RLS is a genetically heterogeneous complex trait with high prevalence but large phenotypic variability, and both idiopathic and secondary forms contribute to prevalence statistics. Thus, fine-mapping and further genetic studies in RLS patients may reveal differential effects of the various risk loci as observed in these mice and in other studies. In future investigations, an RLS-related mutation within *Meis1* may provide a more disease-relevant disruption of Meis1 function in mice and therefore a better RLS phenotype (Allen et al., 2017).

## MATERIALS AND METHODS

### Animals and breeding

The *Meis1^tm1Mtor^* mice used in the experiments were created in Madrid, Spain (Azcoitia et al., 2005) and have been bred on the C57BL/6JOlaHsd (Envigo, Horst, The Netherlands) background. In this strain, *Meis1* has been inactivated by knocking in a modified ERT2 domain. The result is predicted to encode an inactive protein product. The mouse line has been bred in Munich, Germany, backcrossing to wild-type C57BL/6JOlaHsd every generation. Mouse husbandry was performed according to the FELASA recommendations (Nicklas et al., 2002) (http://www.felasa.eu) in a controlled specific pathogen free (SPF) hygiene standard environment. The mice had access to *ad libitum* standard feed and water always. All tests were carried out with approval for the ethical treatment of animals by the responsible authority of the Regierung von Oberbayern (Government of Upper Bavaria).

### Analysis of *Meis1^tm1Mtor^* mice

**Fig. 5.**
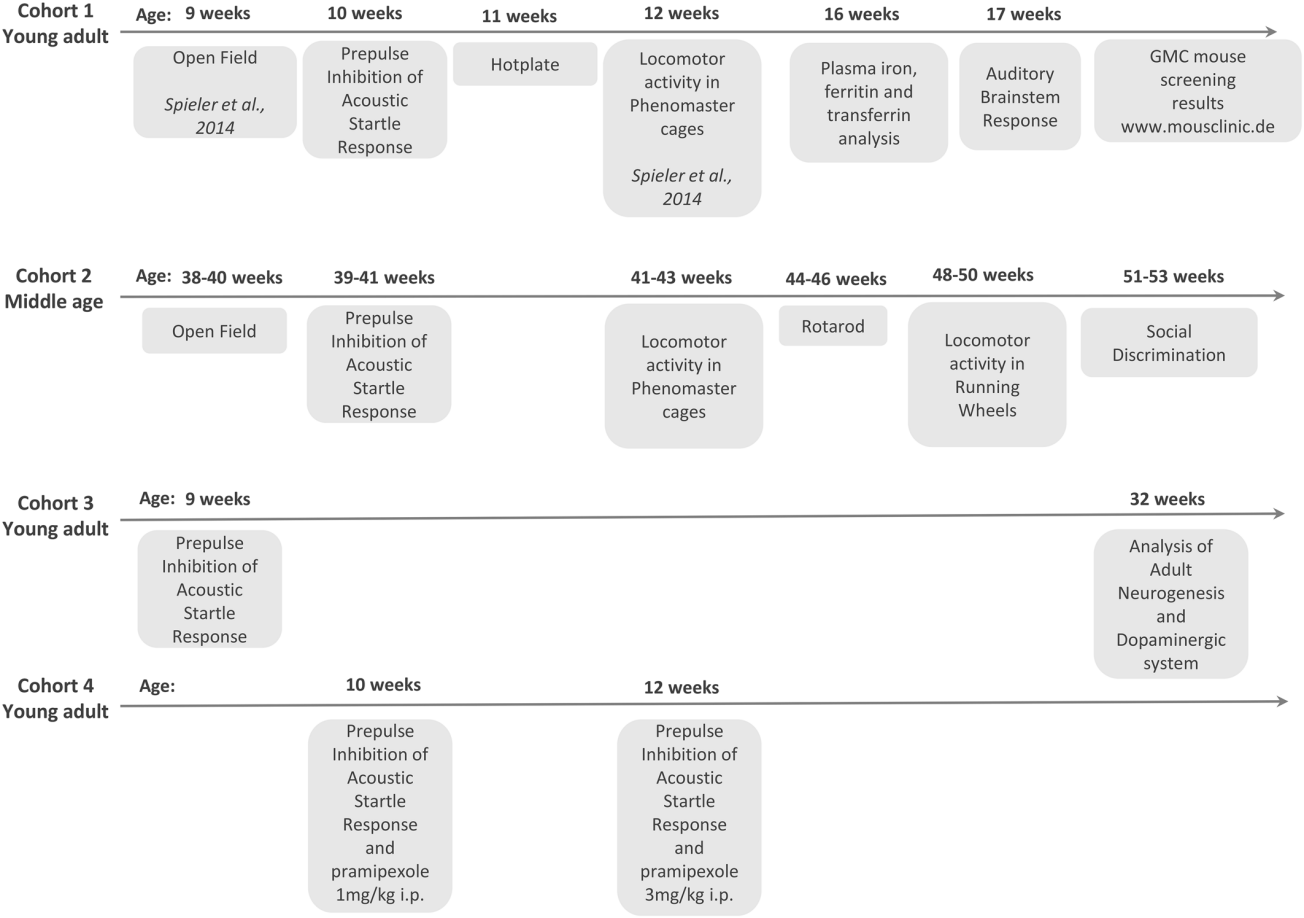
**An overview of the four cohorts of mice used and the age at which each of the tests were performed.** Three young-adult and one middle-aged cohort was used. Results from additional German Mouse Clinic (GMC) screening can be found at the GMC phenomap (www.mouseclinic.de).

Four different mouse cohorts were bred for the experiments described here and the corresponding tests and order performed are shown in Fig. 5. In all experiments, heterozygous animals (*Meis1*^+/−^) were used, homozygotes being embryonic lethal (Azcoitia et al., 2005). The mice from the different cohorts were tested together with their wild-type (*Meis1*^+/+^) littermates as control animals for behavioral/neurological measures as follows: cohort 1 (15 male +/+, 15 male +/−, 15 female +/+ and 15 female +/−) were tested in: open field (at 9 weeks; published in Spieler et al., 2014); prepulse inhibition (at 10 weeks); hotplate (at 11 weeks); locomotor activity in PhenoMaster cages (at 12 weeks; published in Spieler et al., 2014); plasma iron level analysis (at 16 weeks); and ABR (at 17 weeks); cohort 2 (10 male +/−, 13 male +/+, 9 female +/−, 12 female +/+) were tested in: open field (at 38-40 weeks); PPI (at 39-41 weeks); rotarod (at 44-46 weeks); locomotor activity in the PhenoMaster cages (at 41-43 weeks); locomotor activity in running wheels (at 48-50 weeks); and social discrimination (at 51-53 weeks); cohort 3 (15 male +/+, 15 male +/−, 15 female +/+ and 15 female +/−) were tested for: PPI (at 9 weeks), and a subset of these animals were perfused with paraformaldehyde (PFA) and used for brain-tissue stereological analysis of dopaminergic neuron number (TH+ cells) and adult neurogenesis (DCX+ cells). The mice (30 animals per sex per genotype) from cohort 4 were tested for sensitivity to pramipexole in PPI (at 10 weeks for 1 mg/kg body weight and at 11 weeks for 3 mg/kg body weight). Mice from the first cohort also underwent the standardized phenotype screening at the German Mouse Clinic (GMC; www.mouseclinic.de). The test battery included additional screening of the mice in the areas of dysmorphology, clinical chemistry, energy metabolism, cardiovascular studies, eye function, immunology, allergy, steroid metabolism, lung function and pathology. The screens were performed as described in previous publications (Fuchs et al., 2009; Gailus-Durner et al., 2005, 2009) and the results of this analysis can be found on the GMC phenomap (tools.mouseclinic.de/phenomap/jsp/annotation/public/phenomap.jsf).

### Open field

The open-field analysis was carried out as we described previously (Garrett et al., 2012; Hölter et al., 2013; Zimprich et al., 2014). It consisted of a transparent and infrared-light-permeable acrylic test arena with a smooth floor (internal measurements: 45.5×45.5×39.5 cm). Illumination levels were set at approx. 150 lux in the corners and 200 lux in the middle of the test arena. Data were recorded and analyzed using the ActiMot system (TSE, Bad Homburg, Germany).

### PPI of the ASR

Mice were housed with food and water available *ad libitum* under standard laboratory conditions. Animals were separated based on sex, but not genotype. PPI was assessed using a startle apparatus setup (Med Associates Inc., VT, USA) including four identical sound-attenuating cubicles. The protocols were written using the Med Associates ‘Advanced Startle’ software. Experiments were carried out between 08:30 h and 17:00 h. Background noise was 65 dB, and startle pulses were bursts of white noise (40 ms). A session was initiated with a 5-min acclimation period followed by five presentations of leader startle pulses (110 dB) that were excluded from statistical analysis. Trial types for the PPI included four different prepulse intensities (67, 69, 73, 81 dB); each prepulse preceded the startle pulse (110 dB) by a 50-ms inter-stimulus interval. Each trial type was presented ten times in random order, organized in ten blocks, each trial type occurring once per block. Inter-trial intervals varied from 20 to 30 s. This protocol is based on the protocol used in IMPRESS from the IMPC (see www.mousephenotype.org/impress), adapted to the specifications of our startle equipment.

### Drug preparation

Pramipexole (pramipexole dihydrochloride, Sigma Aldrich, St Louis, MO, USA) was prepared in saline and injected at two different doses (1 and 3 mg/kg body weight). For the PPI test with drug treatment, *Meis1* and control mice were assigned to receive either pramipexole (1 and 3 mg/kg body weight) or saline vehicle and this was balanced for genotype, startle chamber and treatment. The mice were tested for PPI 1 h after administration of the treatment and the i.p. injection volume was 10 ml/kg body weight. The experimenter was not blinded during the PPI testing as the test is automated and there was no possibility for observer bias.

### ABR

ABR measurements were performed using an RP2.1 workstation (Tucker-Davis Technologies, FL, USA) as previously described (Lee et al., 2015). In short, animals were anesthetized and electrodes were placed on the vertex (reference) and ventrolateral to the ears (active and ground) of the animals. ABR potentials, as responses to broadband clicks or pure-tone frequencies, were recorded. Frequencies ranged from 6 to 30 kHz with a 6 kHz stepping, and sound pressure levels ranged from 0 to 85 dB with 5 dB steps. Hearing thresholds were determined for each frequency as the lowest sound pressure level (SPL) producing a measurable ABR pattern response.

### Hotplate

In nociception, mice were screened using a hotplate assay. A mouse was placed on a 28-cm-diameter metal surface maintained at 52+0.2°C surrounded by a 20-cm-high Plexiglass wall (TSE, Bad Homburg, Germany). Mice remained for 30 s on the plate or until they performed one of three behaviors regarded as indicative of nociception: hind-paw licking, hind-paw shake/flutter or jumping. The latency of the first sign of pain was compared.

### Social discrimination

The social discrimination procedure consisted of two 4-min exposures of stimulus animals (ovariectomized 129Sv females) to the test animal in a fresh cage to which the test animal had been moved 2 h prior to testing. During the first exposure, one stimulus animal was exposed to the test animal. After a retention interval of 2 h, this stimulus animal was re-exposed to the test animal together with an additional, not previously presented stimulus animal. The duration of investigatory behavior of the test animal towards the stimulus animals was recorded by a trained observer with a hand-held computer. A social recognition index was calculated as time spent investigating the unfamiliar stimulus mouse/time spent investigating both the familiar and unfamiliar stimulus mouse.

### Rotarod

The rotarod (Bioseb, Chaville, France) was used to measure forelimb and hindlimb motor coordination, balance and motor learning ability. The machine was set up in an environment with minimal stimuli such as noise and movement. The rotarod device is equipped with a computer-controlled motor-driven rotating rod. The unit consists of a rotating spindle and five individual lanes, one for each mouse. In general, the mouse is placed perpendicular to the axis of rotation, with head facing the direction of the rotation. All mice were placed on the rotarod at an accelerating speed from 4 to 40 rpm for 300 s with 15 min between each trial. In motor-coordination testing, mice were given four trials at the accelerating speed in one day. The mean latency to fall off the rotarod during the trials was recorded and used in subsequent analysis. Before the start of the first trial, mice were weighed.

### Locomotor activity in PhenoMaster cages

For the evaluation of home-cage locomotor activity, single mice were kept in respirometry cages (PhenoMaster System, TSE Systems, Germany) (Gailus-Durner et al., 2009). The measurement started at 13:00 h (CET) after a 2-h adaptation to the cages and continued until 10:00 h the next morning. The setup allowed the analysis of locomotor activity of individual mice every 20 min, resulting in 63 readings per individual and trial. Two infrared-light-beam frames allowed the monitoring of physical activity (lower frame: distance traveled per 20 min, upper frame: number of rearings per 20 min).

### Voluntary wheel running

All mice were singly housed and provided with an angled running wheel (diameter 15.50 cm) complete with a wireless-controlled activity counter (Wheel Manager software, Med Associates Inc., VT, USA) for 21 days.

### Plasma iron level analysis

Blood samples were collected in the morning at 14 or 15 weeks of age by retrobulbar puncture under isoflurane anesthesia. Determination of clinical chemistry parameters in heparinized plasma samples was done using an AU480 (Beckman-Coulter, Krefeld, Germany) and reagent kits provided by Beckman-Coulter, as described previously (Rathkolb et al., 2013). Hematological analyses were performed with an ABC animal blood counter (Scil animal care company, Viernheim, Germany) on whole-blood samples collected in EDTA-coated tubes.

### Tissue preparation

Animals were deeply anesthetized using CO_2_ and perfused transcardially with 4% PFA in 0.1 M phosphate buffer. Brains were dissected from the skulls, post-fixed overnight in 4% PFA at 4°C and then transferred to a 30% (w/v) sucrose solution until saturated. Brains were then sectioned on a dry-ice-cooled block with a sliding microtome (Leica, Bensheim) into 40-µm-thick coronal free-floating sections and stored at −20°C in a cryoprotectant solution containing 25% ethylene glycol and 25% glycerine in phosphate buffer. A one-in-six series of sections was taken for analysis from the brains of a subset of animals from each group.

### Immunostaining

For immunostaining of DCX and TH, an Avidin-Biotin Complex (ABC) method like that employed previously (Garrett et al., 2012) was used. For the DCX staining, a primary rabbit polyclonal anti-DCX antibody (1:500, Abcam, cat. no: ab18723) was used on this occasion with a biotinylated goat anti-rabbit IgG (1:300; Jackson ImmunoResearch Laboratories Inc.) and 3,3′-diaminobenzidine (DAB) as the chromogen. For the TH staining, a primary rabbit anti-TH antibody (1:1000, Pel-Freeze, cat. no: P40101) was used with a biotinylated goat anti-rabbit IgG (1:300; Jackson ImmunoResearch Laboratories Inc.) and DAB as the chromogen.

### Quantification of TH+ and DCX+ neurons

Estimation of the total number of TH+ and DCX+ cells was determined using a method based on the stereological optical fractionator principle and the semiautomatic StereoInvestigator system (MicroBrightField Inc., Williston, VT, USA). For TH+-cell estimation, the SNpc and VTA were traced in every 6th section. For DCX+-cell estimation, the olfactory bulb granular cell layer (OB) was traced in every 6th section. Immunopositive cells were quantified by systematic random sampling using the following settings: TH+ cells in the SNpc and VTA: a scan grid size of 200×200 µm and a counting frame size of 70×70 µm; DCX+ cells in the OB: a scan grid size of 200×200 µm and a counting frame of 100×100 µm. Cells that intersected the uppermost focal plane or the lateral exclusion borders of the counting frame were not quantified. These settings were sufficient to keep the Gundersen coefficient of error (*m*=1) less than 0.1 for each animal.

### Statistics

Tests for genotype effects were done either by using *t*-test, Wilcoxon rank sum test, linear models or ANOVA. The choice of the method depended on the assumed distribution of the parameter and the nature of the question posed to the data. Correlations in the aged cohort were evaluated using Pearson correlation coefficient. A *P*-value of 0.05 was used as level of significance without correction for multiple testing. The analyses were performed using the programming language R (version 3.0.2).
